# Supplementary material for: The genetic and biochemical basis of human leading strand synthesis
Source: Nat Commun. 2025 Dec 4;17:412. doi: 10.1038/s41467-025-67107-7 (PMC12796321; doi:10.1038/s41467-025-67107-7)
Supplement: Supplementary file 1 — Supplementary Information [file 41467_2025_67107_MOESM1_ESM.pdf]

Supplemental Material

The genetic and biochemical basis of human leading strand synthesis

Alessandro Agnarelli[1*], Lauryn Buckley-Benbow[1*], Meryem Ozgencil[1], Melanie Lad[2], Khamal Kwesi Ampah[2], Alex Kalinka[2], Ondrej Belan[3], Sarah Maslen[4], Mark J. Skehel[4], David Walter[2], Matthew Day[5#] and Roberto Bellelli[1#]

Supplementary Figure 1

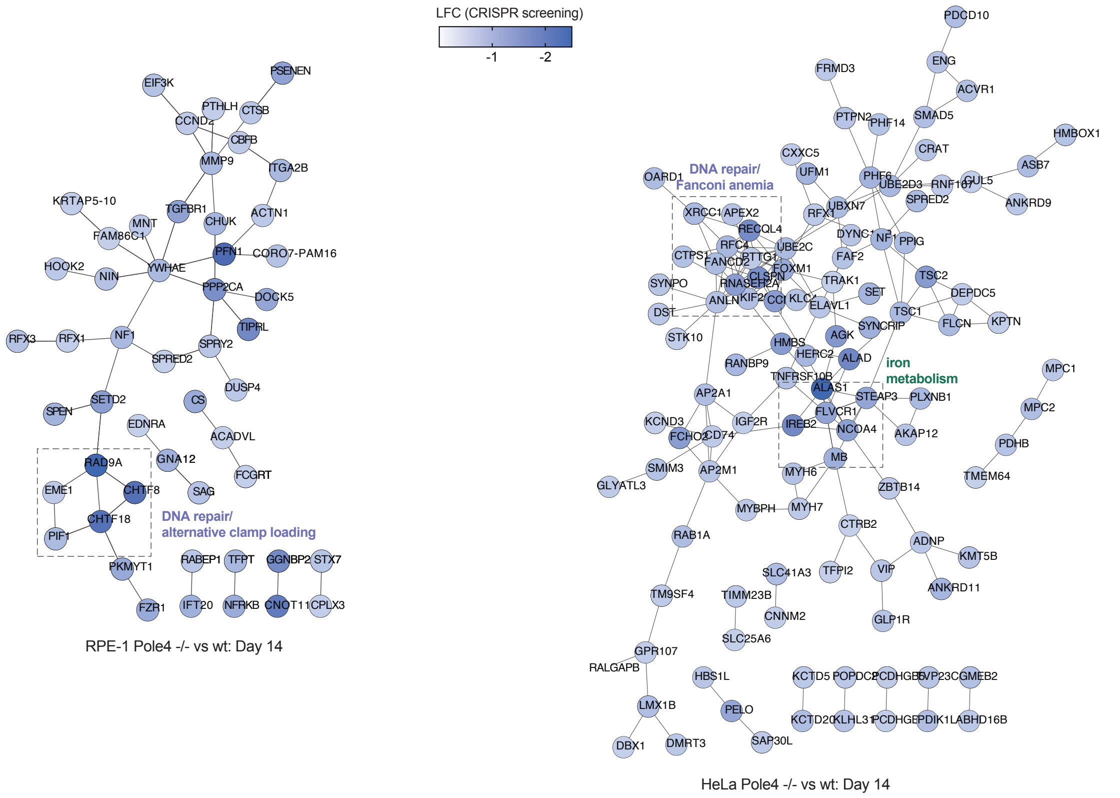

Supplementary Figure 1.

STRING analysis of interactions between statistically significant hits in HTF and RPE1 p53-/- POLE4 KO cells after 13 and 14 days, respectively, of puromycin selection.

Supplementary Figure 2

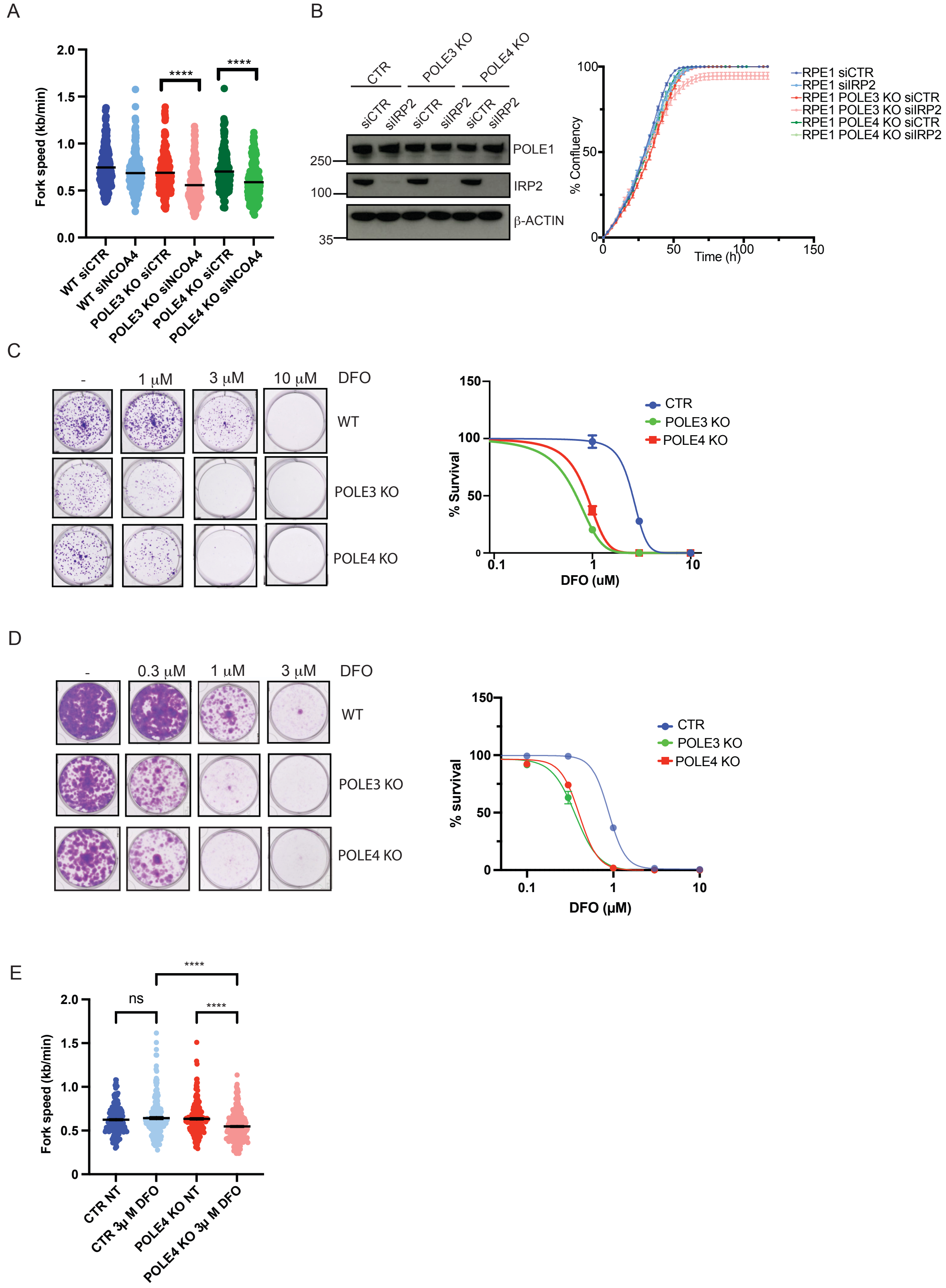

Supplementary Figure 2.

(A) Bar graphs showing replication fork extension rates from HTF WT, POLE3 and POLE4 KO transfected with siRNA against NCOA4 or control; unpaired t-test analysis **** $p<0.0001$. At least 200 fibers were analysed for condition. (B) Left: western blot analysis of IRP2 and POLE1 levels in HTF WT, POLE3 KO and POLE4 KO cells transfected with siRNAs against IRP2 or CTR. β-ACTIN was used for normalization. Right: Change in % confluency of HTF WT, POLE3 KO and POLE4 KO cells transfected with siRNAs against IRP2 or CTR and imaged every 3 hours for 120 hours by IncuCyte Live-cell imaging; results are reported as mean +/- SEM of triplicate biological experiments. (C) Left: representative pictures of crystal violet staining from HTF WT, POLE3 KO and POLE4 KO cells treated with increasing concentrations of Deferoxamine (DFO). Right: quantification of clonogenic survival of HTF WT, POLE3 KO, and POLE4 KO cells treated with increasing concentrations of Deferoxamine (DFO); results are reported as mean +/- SD of triplicate experiments. (D) Left: representative pictures of crystal violet staining from RPE1 p53-/- WT, POLE3 KO and POLE4 KO cells treated with increasing concentrations of Deferoxamine (DFO). Right: quantification of clonogenic survival of RPE1 p53-/- WT, POLE3 KO, and POLE4 KO cells treated with increasing concentrations of Deferoxamine (DFO); results are reported as mean +/- SD of triplicate experiments. (E) Bar graphs showing replication fork extension rates from HTF WT and POLE4 KO treated or not with DFO 3 uM for 48 hours; unpaired t-test analysis **** $p<0.0001$. At least 200 fibers were analysed for condition

Supplementary Figure 3

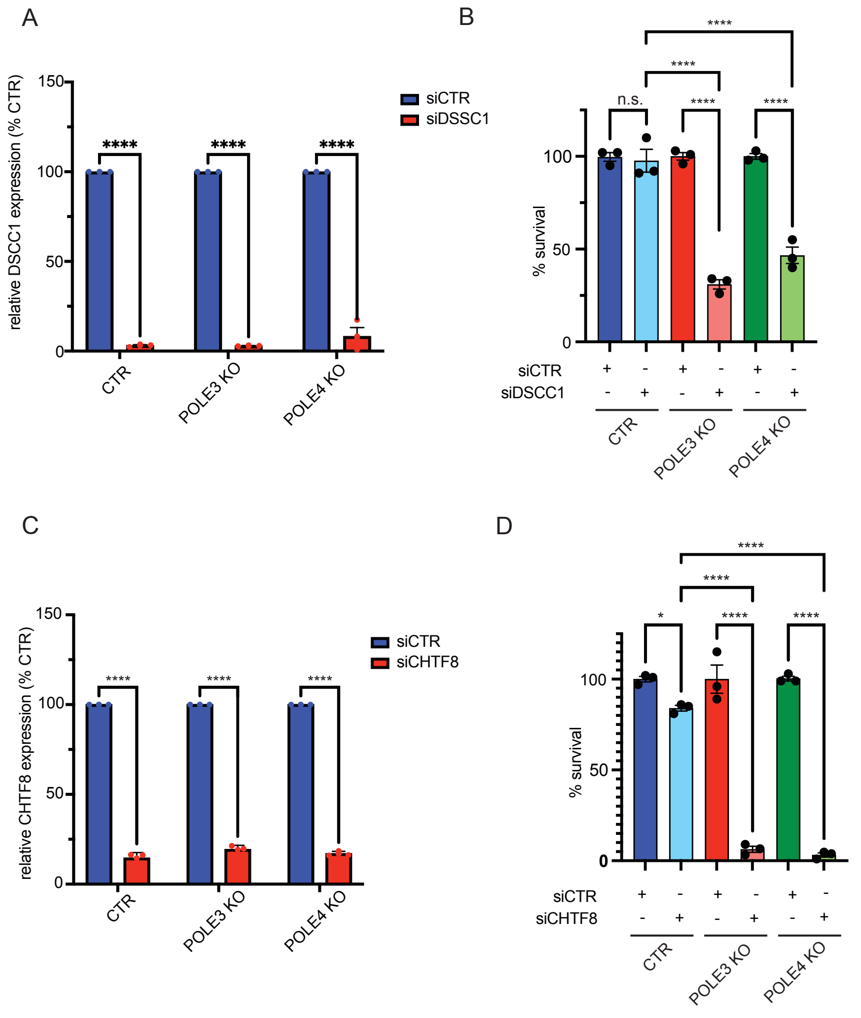

Supplementary Figure 3.

(A) Bar graphs showing relative mRNA expression levels of DSCC1 from HTF WT, POLE3 and POLE4 KO cells transfected with siRNA against DSCC1 or CTR; unpaired t-test analysis: **** p<0.0001. Results were obtained from three biological replicates. (B) Bar graphs showing % survival from Colony Forming Assays (CFAs) of HTF WT, POLE3 and POLE4 KO cells transfected with siRNA against DSCC1 or CTR; unpaired t-test analysis: **** P<0.0001, n.s. not significant. Results were obtained from three biological replicates. (C) Bar graphs showing relative mRNA expression levels of CHTF8 from HTF WT, POLE3 and POLE4 KO cells transfected with siRNA against CHTF8 or CTR. unpaired t-test analysis: **** p<0.0001. results were obtained from three biological replicates. (D) Bar graphs showing % survival from CFAs of HTF WT, POLE3 and POLE4 KO cells transfected with siRNA against DSCC1 or CTR; unpaired t-test analysis: * p<0.05, **** P<0.0001. Results were obtained from three biological replicates.

Supplementary Figure 4

A

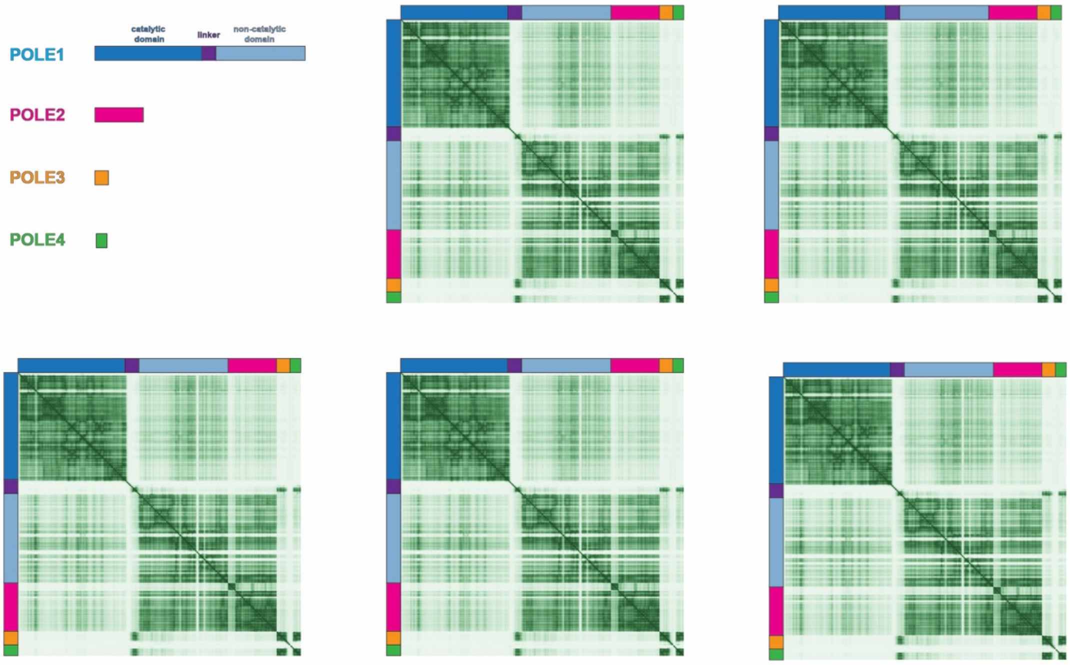

B

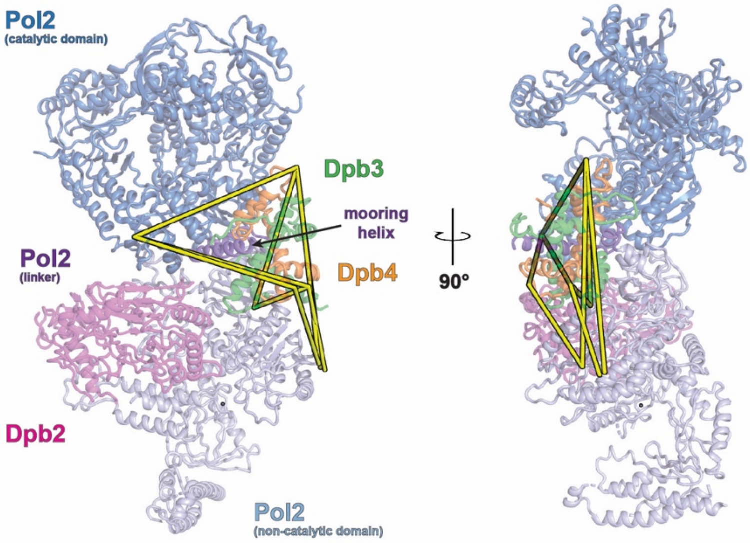

Supplementary Figure 4.

(A) Schematic representation of the protein chains and PAE plots shown for 5 models produced for POLE1, POLE2, POLE3 and POLE4 predicted by AlphaFold3. In all five models the results suggest high degree of flexibility between the two domains of POLE1 and that the mooring helix inside the linker region of POLE1 can be seen to be predicted to interact with the histone folds of POLE3 and POLE4. (B) Orthogonal views of the structure of the rigid confirmation of yeast proteins (PDB: 6WJV), coloured as for human complex with Pol2 shown in separate shades of blue for the catalytic, linker and non-catalytic domains, and Dpb2, Dpb4 and Dpb3 coloured magenta, orange and green respectively. Crosslinks for equivalent residues from human proteins based on Clustal Omega alignments shown as yellow bars. Several crosslinks are either too far to exist in the rigid conformation or cannot occur as the cross linker would have to pass through solid protein to satisfy the distance constraints of the crosslinker

Supplementary Figure 5

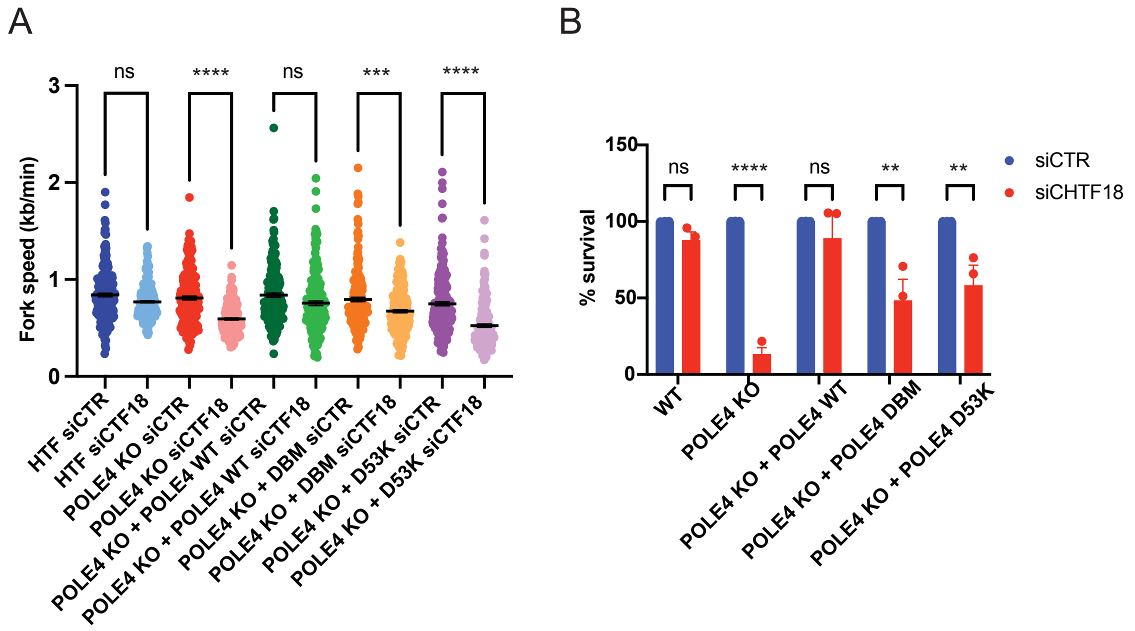

Supplementary Figure 5.

(A) Bar graphs showing replication fork extension rates from HTF WT and POLE4 KO complemented or not with POLE4 WT, the DNA binding mutant (DBM) or the D53K mutant, and transfected with siRNA against CHTF18 or control; unpaired t-test analysis *** p<0.001; **** p<0.0001. (B) Bar graphs showing relative survival from HTF WT and POLE4 KO complemented or not with POLE4 WT, the DBM or the D53K mutant, and transfected with siRNA against CHTF18 or control; unpaired t-test analysis ** p<0.01; **** p<0.0001.

Supplementary Figure 6

A

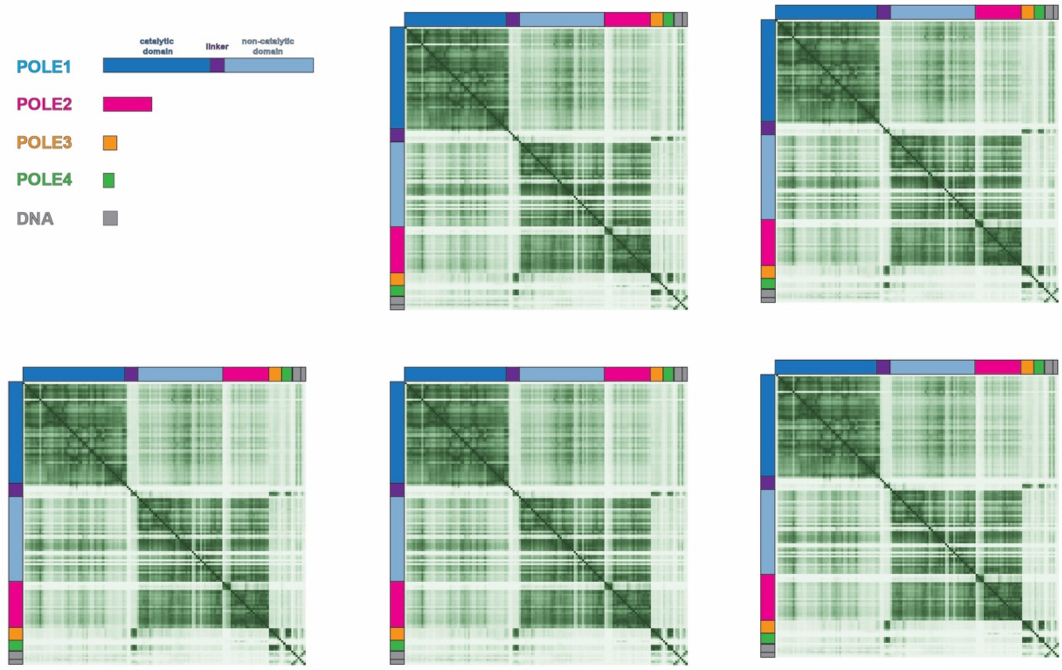

B

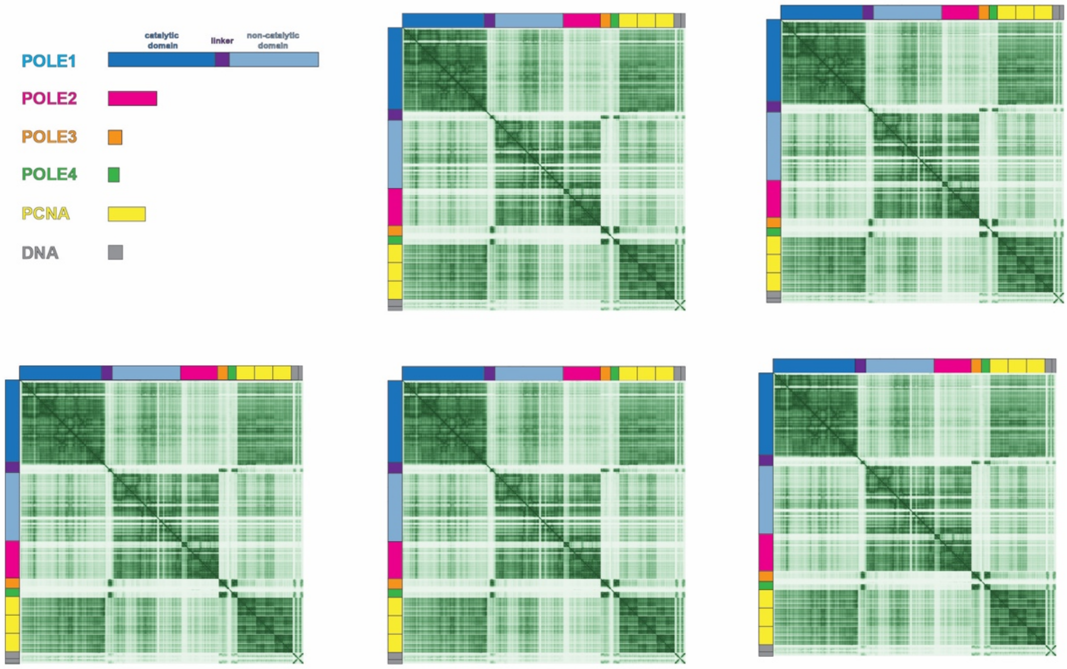

Supplementary Figure 6.

(A) PAE plots are shown for 5 models produced for POLE1, POLE2, POLE3 and POLE4 with a primer template DNA structure predicted by AlphaFold3. In all five models the DNA can be seen to interact with both the catalytic domain of POLE1 and additional interactions made with the POLE3 and POLE4 accessory subunits. (B) PAE plots are shown for 5 models produced for POLE1, POLE2, POLE3 and POLE4 with a primer template DNA structure and three copies of PCNA predicted by AlphaFold3. In all five models the DNA can still be seen to interact with the POLE3 and POLE4 accessory subunits as it emerges from the PCNA ring, which itself makes contacts with the catalytic domain and linker region of POLE1.

Supplementary Figure 7

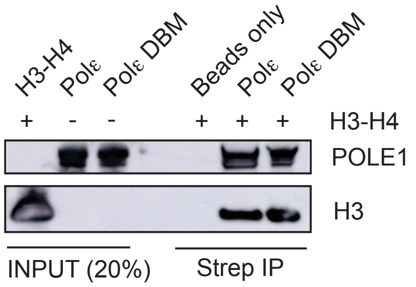

Supplementary Figure 7.

Western blot analysis of streptavidin pull downs of Pol wt ε or DBM incubated with histone H3-H4. Membranes were incubated with the indicated antibodies.

Supplementary Figure 8

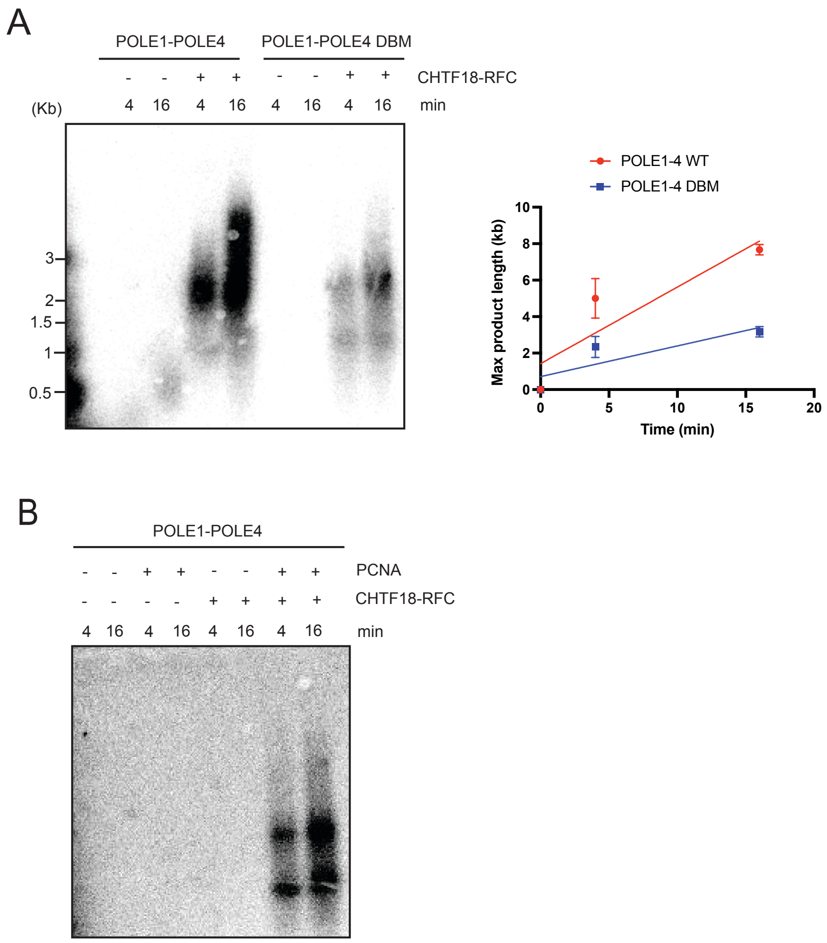

Supplementary Figure 8.

(A) Left: Representative radioactive primer extension assay performed on M13mp18 substrates in the presence of the indicated proteins and for the indicated time points. PCNA and RPA were included in all the reactions. Right: quantification of triplicate radioactive primer extension assays performed with WT Polε or DB mutant. (B). Representative radioactive primer extension assay performed on M13mp18 substrates in the presence of the indicated proteins and for the indicated time points.

Supplementary Figure 9

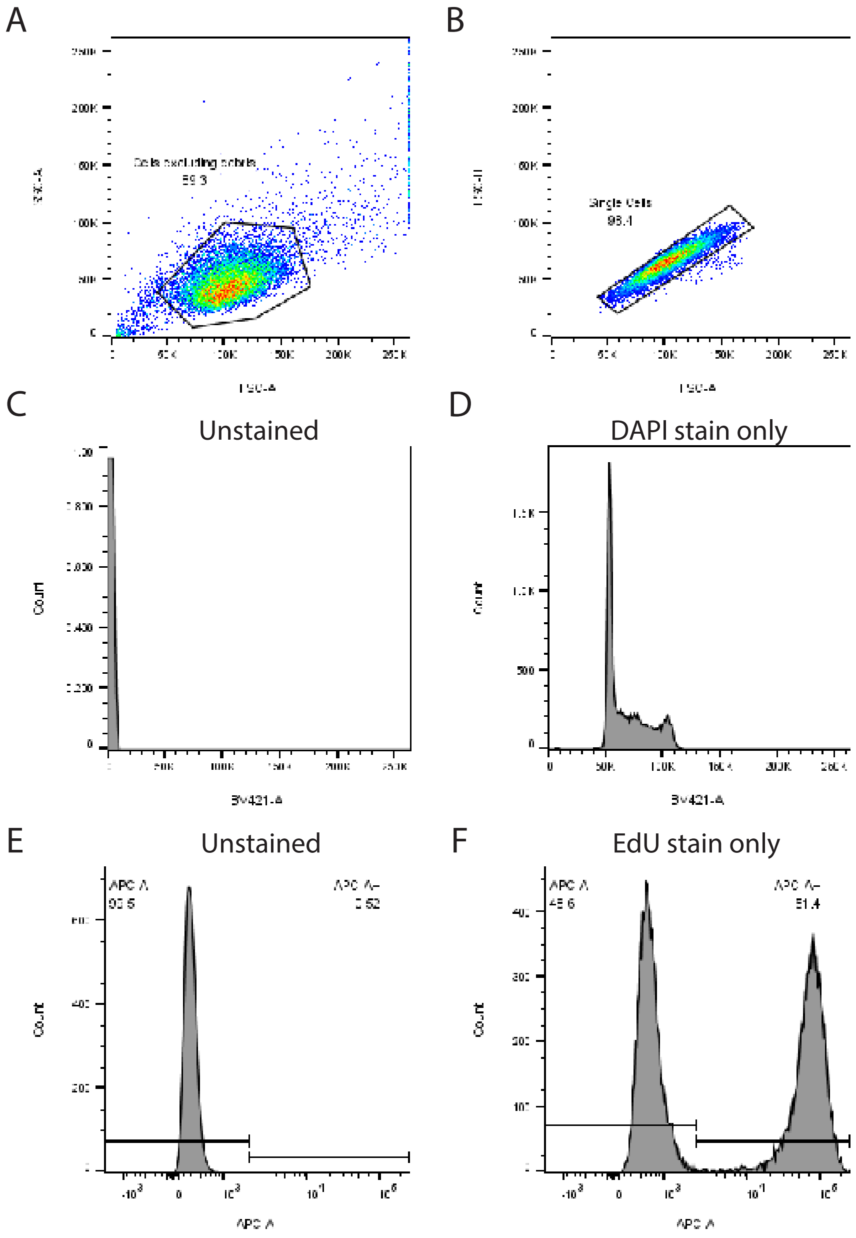

Supplementary Figure 9.

(A) Cells were first gated to exclude debris by drawing a tight polygon around the population on a density dot plot with X-axis as FSC-A and Y-axis as SSC-A. (B) The cells population excluding debris was then plotted on a density dot plot with X-axis as FSC-A and Y-axis as FSC-H to draw a gate around the singlet cell population. To gate for DAPI positive cells, we checked an (C) unstained population against a (D) DAPI only stained population. To gate for EdU-positive cells, we compared an (E) unstained population against an (F) EdU only stained population and gated for EdU-negative versus EdU-positive cells.
