# Supplementary material for: The genetic and biochemical basis of human leading strand synthesis
Source: Nat Commun. 2025 Dec 4;17:412. doi: 10.1038/s41467-025-67107-7 (PMC12796321; doi:10.1038/s41467-025-67107-7)
Supplement: Supplementary file 2 — Descriptions of Additional Supplementary Files [file 41467_2025_67107_MOESM2_ESM.pdf]

Description of Additional Supplementary Files

Supplementary Data 1. A gene list of high-confidence CRISPR drop-out hits ($p<0.05$; $L2FC<-0.5$) for each of the screening conditions.

Supplementary Data 2. List of crosslinks identified between Pol epsilon subunits in the cross-linking mass spectrometry experiment.
