# Supplementary material for: The genetic and biochemical basis of human leading strand synthesis
Source: Nat Commun. 2025 Dec 4;17:412. doi: 10.1038/s41467-025-67107-7 (PMC12796321; doi:10.1038/s41467-025-67107-7)
Supplement: Supplementary file 5 — Reporting Summary [file 41467_2025_67107_MOESM5_ESM.pdf]

nature portfolio

Corresponding author(s): Roberto Bellelli

Last updated by author(s): Apr 28, 2025

Reporting Summary

Nature Portfolio wishes to improve the reproducibility of the work that we publish. This form provides structure for consistency and transparency in reporting. For further information on Nature Portfolio policies, see our Editorial Policies and the Editorial Policy Checklist.

Statistics

For all statistical analyses, confirm that the following items are present in the figure legend, table legend, main text, or Methods section.

n/a	Confirmed	
☐	☒	The exact sample size (n) for each experimental group/condition, given as a discrete number and unit of measurement
☐	☒	A statement on whether measurements were taken from distinct samples or whether the same sample was measured repeatedly
☐	☒	The statistical test(s) used AND whether they are one- or two-sided<br>*Only common tests should be described solely by name; describe more complex techniques in the Methods section.*
☒	☐	A description of all covariates tested
☒	☐	A description of any assumptions or corrections, such as tests of normality and adjustment for multiple comparisons
☐	☒	A full description of the statistical parameters including central tendency (e.g. means) or other basic estimates (e.g. regression coefficient) AND variation (e.g. standard deviation) or associated estimates of uncertainty (e.g. confidence intervals)
☐	☒	For null hypothesis testing, the test statistic (e.g. F, t, r) with confidence intervals, effect sizes, degrees of freedom and P value noted<br>*Give P values as exact values whenever suitable.*
☒	☐	For Bayesian analysis, information on the choice of priors and Markov chain Monte Carlo settings
☒	☐	For hierarchical and complex designs, identification of the appropriate level for tests and full reporting of outcomes
☒	☐	Estimates of effect sizes (e.g. Cohen's d, Pearson's r), indicating how they were calculated

Our web collection on statistics for biologists contains articles on many of the points above.

Software and code

Policy information about availability of computer code

Data collection	N/A
Data analysis	all analysis were performed using Prism 10

For manuscripts utilizing custom algorithms or software that are central to the research but not yet described in published literature, software must be made available to editors and reviewers. We strongly encourage code deposition in a community repository (e.g. GitHub). See the Nature Portfolio guidelines for submitting code & software for further information.

Data

Policy information about availability of data

All manuscripts must include a data availability statement. This statement should provide the following information, where applicable:

- Accession codes, unique identifiers, or web links for publicly available datasets
- A description of any restrictions on data availability
- For clinical datasets or third party data, please ensure that the statement adheres to our policy

Further information and requests for reagents should be directed and will be fulfilled by the Lead contact, Roberto Bellelli

Research involving human participants, their data, or biological material

Policy information about studies with human participants or human data. See also policy information about sex, gender (identity/presentation), and sexual orientation and race, ethnicity and racism.

Reporting on sex and gender	N/A
Reporting on race, ethnicity, or other socially relevant groupings	N/A
Population characteristics	N/A
Recruitment	N/A
Ethics oversight	N/A

Note that full information on the approval of the study protocol must also be provided in the manuscript.

Field-specific reporting

Please select the one below that is the best fit for your research. If you are not sure, read the appropriate sections before making your selection.

☒ Life sciences ☐ Behavioural & social sciences ☐ Ecological, evolutionary & environmental sciences

For a reference copy of the document with all sections, see nature.com/documents/nr-reporting-summary-flat.pdf

Life sciences study design

All studies must disclose on these points even when the disclosure is negative.

Sample size	sample size was calculated based on the specific experiment to ensure enough statistical power
Data exclusions	No data were excluded
Replication	experiments were performed at least in biological triplicate
Randomization	N/A
Blinding	N/A

Reporting for specific materials, systems and methods

We require information from authors about some types of materials, experimental systems and methods used in many studies. Here, indicate whether each material, system or method listed is relevant to your study. If you are not sure if a list item applies to your research, read the appropriate section before selecting a response.

Materials & experimental systems

n/a	Involved in the study	
☐	☒	Antibodies
☐	☒	Eukaryotic cell lines
☒	☐	Palaeontology and archaeology
☒	☐	Animals and other organisms
☒	☐	Clinical data
☒	☐	Dual use research of concern
☒	☐	Plants

Methods

n/a	Involved in the study	
☒	☐	ChIP-seq
☐	☒	Flow cytometry
☒	☐	MRI-based neuroimaging

Antibodies

Antibodies used	Thermo Fisher antibodies: Goat anti-rat IgG (H+L) Antibody, Alexa Fluor 594 Conjugated (#A-11007, FACS), Goat anti-rat IgG (H+L) Antibody, Alexa Fluor 594 Conjugated (#A-11059, Fiber), Goat anti-Rabbit IgG (H+L) Cross-Adsorbed Secondary Antibody, Alexa Fluor™ 488 (#A-11008, FACS), Peroxidase-conjugated Goat anti-Mouse IgG (H+L) (G-21040, IP), Peroxidase-conjugated Goat anti-Rabbit IgG (H+L) (G-21234, IP). Antibody from Bellelli et al,. Mol Cell 2018a: Rabbit polyclonal anti-POLE4 (WB, IP). Antibody from

	Bellelli et al., Mol Cell 2014: Rabbit polyclonal anti-NCOA4. Antibodies from Bethyl: Rabbit polyclonal anti-POLE3 (A301-245A, WB, IP), Rabbit polyclonal anti-CHTF18 (A301-883A, WB), Rabbit polyclonal anti-POLD1 (A304-007A, WB). Antibodies from Santa Cruz: Mouse monoclonal anti-IRP2 (sc-33682, WB), Mouse monoclonal anti-PCNA (sc-56). Antibodies from Cell Signalling Technology: Rabbit polyclonal anti phospho-histone H2AX (Ser139) (2577S, FACS), Rabbit monoclonal anti-beta-actin (4970S, WB). Antibody from Sigma-Aldrich: Mouse Monoclonal anti-tubulin (T6074, WB). Antibody from Abcam: Rat monoclonal anti-BrdU (ab6326, Fiber). Antibody from Becton Dickinson: Mouse monoclonal anti-BrdU (347580, Fiber). Antibody from Genetex: Rabbit polyclonal anti-POLE (GTX132100, WB, IP).
Validation	Primary antibodies were validated by using siRNA and shown via the manufacturer's website.

Eukaryotic cell lines

Policy information about cell lines and Sex and Gender in Research

Cell line source(s)	Cell lines from Hill, Ozgencil et al., Cell Reports 2024: HeLa TRex Flip In sgCTR, HeLa TRex Flip In sgPOLE3 (POLE3 KO), HeLa TRex Flip In sgPOLE4 (POLE4 KO). Cells generated as part of this study: HeLa TRex Flip In sgCHTF19 clone #1, clone #4 and clone #6. Cells from Hustedt et al., 2019: RPE1 p53-/- CTR, RPE1 p53 -/- sgPOLE3 (POLE3 KO), RPE1 p53-/- sgPOLE4 (POLE4 KO)
Authentication	Cell lines were STR profiled.
Mycoplasma contamination	All cell lines were regularly tested for Mycoplasma contamination and were negative.
Commonly misidentified lines (See ICLAC register)	N/A

Plants

Seed stocks	N/A
Novel plant genotypes	N/A
Authentication	N/A

Flow Cytometry

Plots

Confirm that:

☒ The axis labels state the marker and fluorochrome used (e.g. CD4-FITC).

☒ The axis scales are clearly visible. Include numbers along axes only for bottom left plot of group (a 'group' is an analysis of identical markers).

☒ All plots are contour plots with outliers or pseudocolor plots.

☒ A numerical value for number of cells or percentage (with statistics) is provided.

Methodology

Sample preparation	Sample preparation is described with more detail in the methods of this manuscript. Cells were prepared and fixed according to the EdU Click-iT kit instructions from Thermo Fisher.
Instrument	LSR Fortessa 3
Software	Samples were collected using the BD FACSDIva software and data were analysed on FlowJo v10
Cell population abundance	Cells excluding debris accounted for 80-90% of events collected. Singlet cells accounted for 83-92% events from the cells excluding debris gate.
Gating strategy	Cells were first gated to exclude debris from the samples using FCS versus SSC dot plot. This gated population of cells was then applied to a graph of FSC-A versus FSC-H and a gate was applied around the singlet population. This singlet population was subsequently applied to all graphs of interest. To determine negative and positive staining, single stains were performed for each experiment and compared against an unstained population to determine the gate for positive staining.

☒ Tick this box to confirm that a figure exemplifying the gating strategy is provided in the Supplementary Information.
